# Analysis of Three-Dimensional Bone Microarchitecture of the Axis Exposes Pronounced Regional Heterogeneity Associated with Clinical Fracture Patterns

**DOI:** 10.1007/s00223-023-01070-7

**Published:** 2023-02-24

**Authors:** Leon-Gordian Koepke, Simon von Kroge, Annika Heuer, Anna Lena Kammal, Benjamin Ondruschka, Tim Rolvien, Lennart Viezens

**Affiliations:** 1 Department of Trauma and Orthopedic Surgery, University Medical Center Hamburg-Eppendorf, Hamburg, Germany; 2 Department of Osteology and Biomechanics, University Medical Center Hamburg-Eppendorf, Hamburg, Germany; 3 Mildred Scheel Cancer Career Center HaTriCS4, University Medical Center Hamburg-Eppendorf, Hamburg, Germany; 4 Institute of Legal Medicine, University Medical Center Hamburg-Eppendorf, Hamburg, Germany

**Keywords:** Odontoid fracture, Spine, HR-pQCT, BMD, Aging

## Abstract

The odontoid process (dens) of the second cervical vertebra (axis) is prone to fracture. While the importance of its skeletal integrity has been previously noted, representative three-dimensional microarchitecture analyses in humans are not available. This study aimed to determine the bone microarchitecture of the axis using high-resolution quantitative computed tomography (HR-pQCT) and to derive clinical implications for the occurrence and treatment of axis fractures. For initial clinical reference, the apparent density of the axis was determined based on clinical computed tomography (CT) images in patients without and with fractures of the axis. Subsequently, 28 human axes (female 50%) obtained at autopsy were analyzed by HR-pQCT. Analyses were performed in three different regions corresponding to zones I (tip of dens), II (base of dens), and III (corpus axis) of the Anderson and D’Alonzo classification. Lower apparent densities based on clinical CT data were detected in zone II and III compared to zone I in both the group without and with fracture. In the autopsy specimens, cortical thickness and bone volume fraction decreased continuously from zone I to zone III. Trabecular and cortical tissue mineral density was lowest in zone III, with no differences between zones I and II. In conclusion, our clinical and high-resolution ex vivo imaging data highlight a marked regional heterogeneity of bone microarchitecture, with poor cortical and trabecular properties near the dens base. These results may partly explain why zones II and III are at high risk of fracture and osteosynthesis failure.

**Supplementary Information:**

The online version contains supplementary material available at 10.1007/s00223-023-01070-7.

## Introduction

The second cervical vertebra, called the axis, contains a prominent odontoid process, the dens axis (DAX). The DAX is prone to fracture especially in elderly patients with compromised bone mineral density. In clinical practice, the Anderson and D’Alonzo fracture classification has become the most commonly used system for axis fractures [1]. In short, type I fractures of the axis (DFTI) occur in the region of the tip of the DAX. Type II fractures (DFTII) extend through the base of the DAX, *i.e.*, the transition from DAX to the corpus axis (CAX). Fractures affecting the CAX are classified as type III fractures (DFTIII). The most common fracture type is the DFTII [2–5]. Especially in elderly patients, DFTII fractures frequently occur in the context of low-energy trauma such as falls from standing height [6, 7]. Due to the demographic change with rapidly increasing numbers of active persons of high age, axis fractures (often also termed odontoid fractures) play an increasingly important role in clinical practice and are associated with a high morbidity and mortality rate [7–9].

Although there is a consensus in the literature that axis fractures must be treated quickly to reduce morbidity and mortality and prevent further complications, there is little knowledge on whether conservative or surgical therapy should be preferred [4, 8–10]. Overall, surgical therapy appears to achieve higher rates of fracture healing for patients > 65 years of age [9, 10]. A commonly applied surgical concept is the anterior screw fixation according to Böhler et al. [11]. However, particularly in patients with limited bone mineral density it is not uncommon for the inserted screws to loosen [12–14] resulting in persistent instability, causing pseudarthrosis with associated pain and risk of myelopathy with neurologic dysfunction [5]. Typically, loosening of the screw takes place at the base of the DAX and the CAX. In this regard, the bone quality and stability resulting from the bone microarchitecture seem to be of paramount importance.

In the past, few studies have been conducted to investigate the bone microarchitecture of the axis and to derive implications for the occurrence and treatment of fractures. In 1994, Amling et al. investigated the microarchitecture of the axis using histological sagittal sections from 22 cadaveric specimens. By determining the bone volume per tissue volume (BV/TV), the trabecular bone pattern factor, and the cortical thickness, a region of least resistance at the base of the DAX was identified in this study [15]. In 1995, the same group also concluded, that there is an increased risk of fractures and subsequent non-unions in osteoporotic bone, based on a reduced trabecular bone mass and a reduced trabecular interconnection at the base of the DAX [16]. However, these studies were based on the sole evaluation of the microarchitecture of the axis based on sagittal two-dimensional sections. Further three-dimensional analyses focused on the occurrence of the residual subdental synchondrosis [17–21].

Although other studies have also been performed to investigate the trabecular bone microarchitecture of the axis in three dimensions [22, 23], a structured analysis in a representative study group has been lacking. Therefore, the current study aims to analyze the bone microarchitecture of the axis using high-resolution quantitative computed tomography (HR-pQCT), to find correlations in clinical specimens analyzed via classical computed tomography (CT), and to derive implications for the occurrence and care of fractures and treatment of the DAX.

## Materials and Methods

### Computed Tomography (CT)

**Fig. 1:**
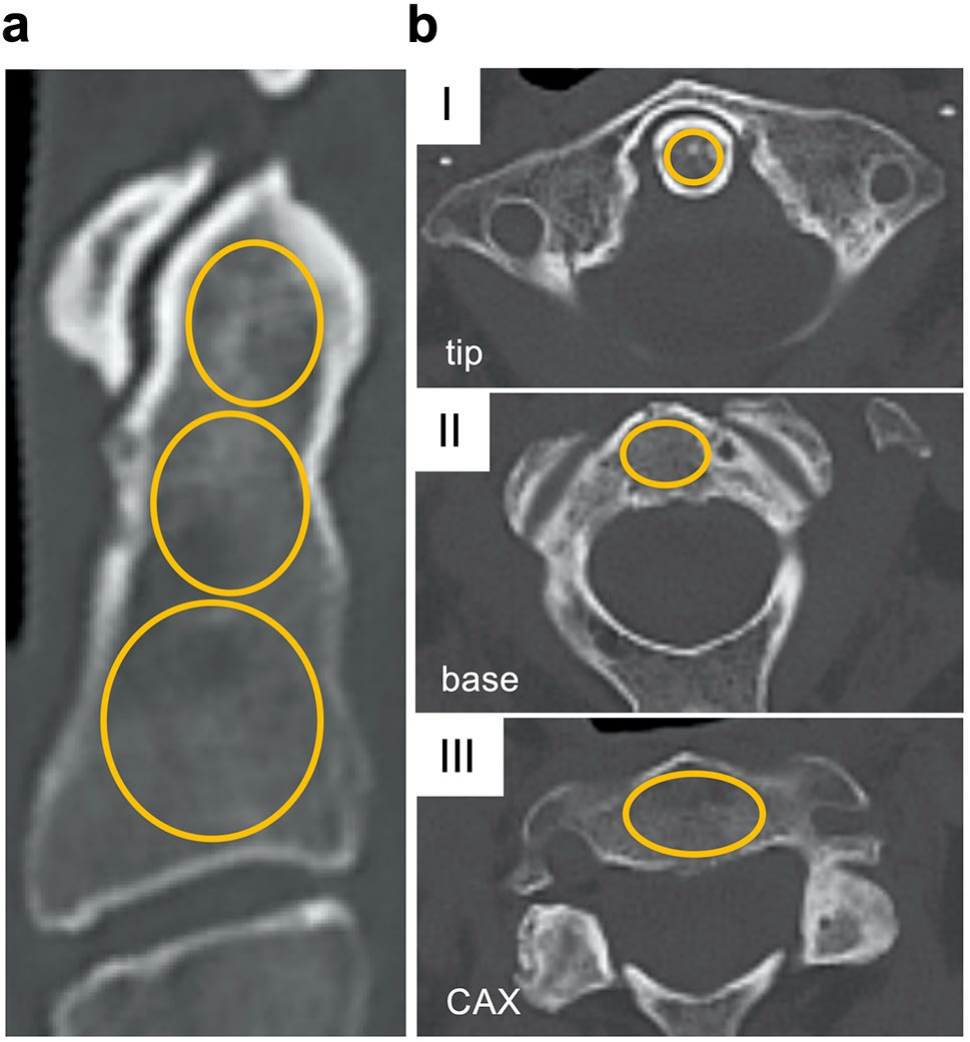
Illustration of clinical CT analysis indicating the volumes of interest (VOI) in each zone. **a** A sagittal section plane through an axis from the non-fracture group is shown. Orange circles indicate the respective VOI in zones I (tip), II (base), and III (CAX). **b** ROI of individual zones determined in axial section planes through the axis

For initial clinical reference, CT scans from *n* = 20 (10 male and 10 female) individuals without fractures of the axis were retrospectively evaluated for apparent density of the axis expressed in Hounsfield units (HU). Only patients without fracture, tumors, or previous surgery in the upper cervical spine were included for analysis. In addition, patients with axis fractures who underwent surgery at our institution between 01/01/2014 and 12/31/2020 were retrospectively analyzed for fracture type. Seventy-eight patients could be identified. In 29 patients, preoperative CT scans of the cervical spine were available. Three patients were excluded due to the presence of spinal osseous metastases or cystic erosion of the DAX. Thus, the apparent density of *n* = 26 (12 male and 14 female) fractured axes was evaluated in preoperative CT scans. In all analyses, a dual-source SOMATOM Force system (Siemens Healthineers, Munich, Germany) or predecessor model was used. Sectioning was performed in all CT scans at a spatial resolution of 1 mm axial thickness and calibration was performed as part of daily routine clinical practice. Multiplanar reconstruction was performed in axial slices. Measurements were performed utilizing the software Centricity Universal Viewer (v6.0, GE Healthcare, Chicago, USA). To evaluate the apparent density corresponding to clinical fracture sites, three zones (zone I-III) were defined according to the fracture classification of Anderson and D’Alonzo (Fig. 1). In detail, zone I was defined as the craniocaudal extent from the most cranial point of the DAX to the inferior border of the anterior arch of the atlas. Zone II extends caudally from this boundary to the transition of the DAX into the CAX. Zone III extends caudally from this boundary to the base plate of the axis. Measurements were performed in sagittal and axial planes. For this purpose, an elliptical region of interest with a diameter as large as possible within the cancellous bone was analyzed in each zone and plane, excluding the cortical bone.

### Collection of Specimens During Autopsy

Human axis specimens (*n* = 28; 14 male and 14 female) were obtained during autopsies and fresh-frozen until further analysis after harvesting. Approval to collect the samples was obtained from the relatives of the deceased. Age, sex, and weight of the individuals were documented. Only anatomically normal and unaltered axis specimens were included, while exclusion criteria included a history of fracture or surgery in the cervical spine or tumor disease. All specimens were anonymized before further analysis. This study was approved by the local ethics committee (2021-300002-WF).

### High Resolution Quantitative Computed Tomography (HR-pQCT)

The fresh-frozen axes were subsequently scanned and analyzed using second-generation HR-pQCT (XTremeCT II, Scanco Medical, Brüttisellen, Switzerland). All samples were scanned using a tube voltage of 68 kV, a current of 1.47 mA and with a spatial resolution of 62 µm. Subsequent analysis of the reconstructed data was performed using the integrated software by Scanco Medical (v6.6). In more detail, the analysis of cortical and trabecular bone was performed for the whole analyzed volume (CC), as well as separately for zones I (tip), II (base), and III (CAX), following the classification according to Anderson and D’Alonzo. The craniocaudal extent of the cylinders corresponded to the above-mentioned limits of the respective zone (I–III). The trabecular bone microarchitecture of the axis was analyzed in a cylindrical volume of interest between the apex of the DAX and the bottom plate of the CAX resulting from the extension of the DAX caudally, based on fracture morphology and the normal screw position of a Böhler screw. Since the DAX has different diameters over its course, the maximum diameter of the DAX was determined at its base. To prevent the inclusion of cortical bone in the spongious cylinders, a diameter of 35% of the maximum diameter of the DAX was defined for the cylinder in zone I, and a diameter of 90% of the maximum diameter of the DAX was defined for the cylinders in zones II and III. The following parameters were determined: BV/TV, number of trabeculae (Tb.N, 1/mm), trabecular thickness (Tb.Th, mm), trabecular separation (Tb.Sp, mm), connectivity density (Conn.D, 1/mm^3^), structure model index (SMI), degree of anisotropy (DA), volumetric (apparent) bone mineral density (vBMD, mg HA/cm^3^), and tissue mineral density (TMD, mg HA/cm^3^), which is defined as the mineral density of mineralized tissue. The latter two were evaluated in trabecular and cortical bone, separately. Further, cortical thickness (Ct.Th, mm) and porosity (Ct.Po, 1) were determined.

### Statistical Analysis

Statistical analysis was performed using SPSS 27.0 (IBM Corp., Armonk, NY, USA). All data is presented as mean ± standard deviation. The Shapiro–Wilk test was used to test for normal distribution. To test for differences between zones Repeated Measures ANOVA (RM ANOVA) with a Geisser-Greenhouse correction in the absence of sphericity and subsequent Tukey post-hoc testing was used. To test for differences between the non-fracture and fracture group, the unpaired two-sided t-test was used. The influence of age and sex on the microstructural evaluation was analyzed with RM ANCOVA testing. The significance level was set at *p* < 0.05.

## Results

### Clinical Fracture Patterns and Apparent Density Assessed by Classical Computed Tomography (CT)

**Fig. 2:**
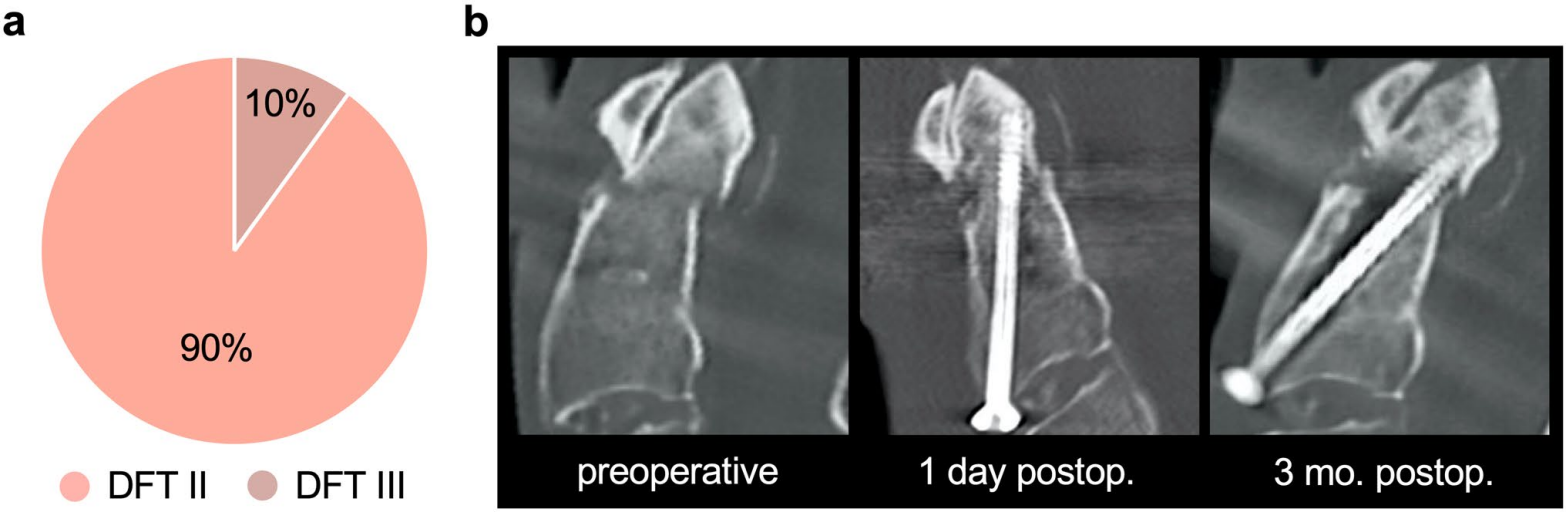
Clinical characteristics of axis fractures. **a** Distribution of fracture types in patients treated surgically at our institution (*n* = 78). DFT II-Anderson and D'Alonzo fracture type II, DFT III–Anderson and D’Alonzo fracture type III. **b** Left panel: Preoperative sagittal reformats of CT images showing a dens axis fracture type II according to Anderson and D’Alonzo (DFTII). Middle panel: Postoperative result after osteosynthesis using a Böhler screw. Right panel: Follow up after 3 months postoperatively with insufficient bone healing and pseudarthrosis accompanied by screw loosening

Analysis of fracture types according to the Anderson and D’Alonzo classification in 78 patients treated surgically at our institution showed that 90% and 10% were DFTII fractures and DFTIII fractures, respectively (Fig. 2a). Notably, DFTII fractures also frequently exhibit osseous complications in terms of nonunion (*i.e.*, pseudarthrosis) (Fig. 2b).

In patients without axis fractures (mean age 81.6 ± 9.2 years), apparent density in zones II and III was lower compared to zone I (zone I *vs*. II *p* < 0.001; zone I vs. III *p* < 0.001; zone II *vs.* III *p* = 0.995) (Table 1). In patients with axis fractures (mean age 81.9 ± 9.9 years), a similar pattern with higher density in zone I compared to zones II and III was detected (zone I *vs.* II *p* < 0.001; zone I *vs.* III *p* < 0.001; zone II *vs.* III *p* = 0.065). In addition, the sagittal measurement of zone II in the fracture collective showed a higher density than the sagittal measurement in zone III (zone II *vs*. III *p* = 0.031). No differences were detected between non-fractured and fractured individuals, neither within the whole dens nor each individual zone.

**Table 1:** Hounsfield-units (HU) in three zones of the axis determined by classical computed tomography (CT) in patients without fracture (non-fracture) and with fracture of the axis

	CC	Zone I (tip)	Zone II (base)	Zone III (CAX)
Hounsfield-units (sagittal)				
Non-fracture	359.8 ± 160.8	568.2 ± 256.5	264.1 ± 164.6*	247.1 ± 93.8^§^
Fracture	333.6 ± 141.1	487.8 ± 243.9	275.5 ± 111.6*	237.7 ± 102.6^§,$^
Hounsfield-units (axial)				
Non-fracture	330.5 ± 155.8	514.7 ± 264.1	231.9 ± 162.1*	244.9 ± 94.4^§^
Fracture	340.0 ± 150.5	484.5 ± 232.5	283.9 ± 150.4*	251.7 ± 113.8^§^
Hounsfield-units (pooled)				
Non-fracture	345.1 ± 157.3	541.5 ± 257.0	248.0 ± 161.8*	246.0 ± 92.1^§^
Fracture	336.8 ± 144.7	486.2 ± 235.9	279.7 ± 124.3*	244.7 ± 104.1^§^

Data is shown as mean ± standard deviation

*CC* complete cylinder

*Zone I vs. II = *p* < 0.05, ^§^zone I vs. III = *p* < 0.05, ^$^zone II vs. III = *p* < 0.05

### Region-Specific Differences in Bone Microarchitecture via* HR-pQCT*

**Fig. 3:**
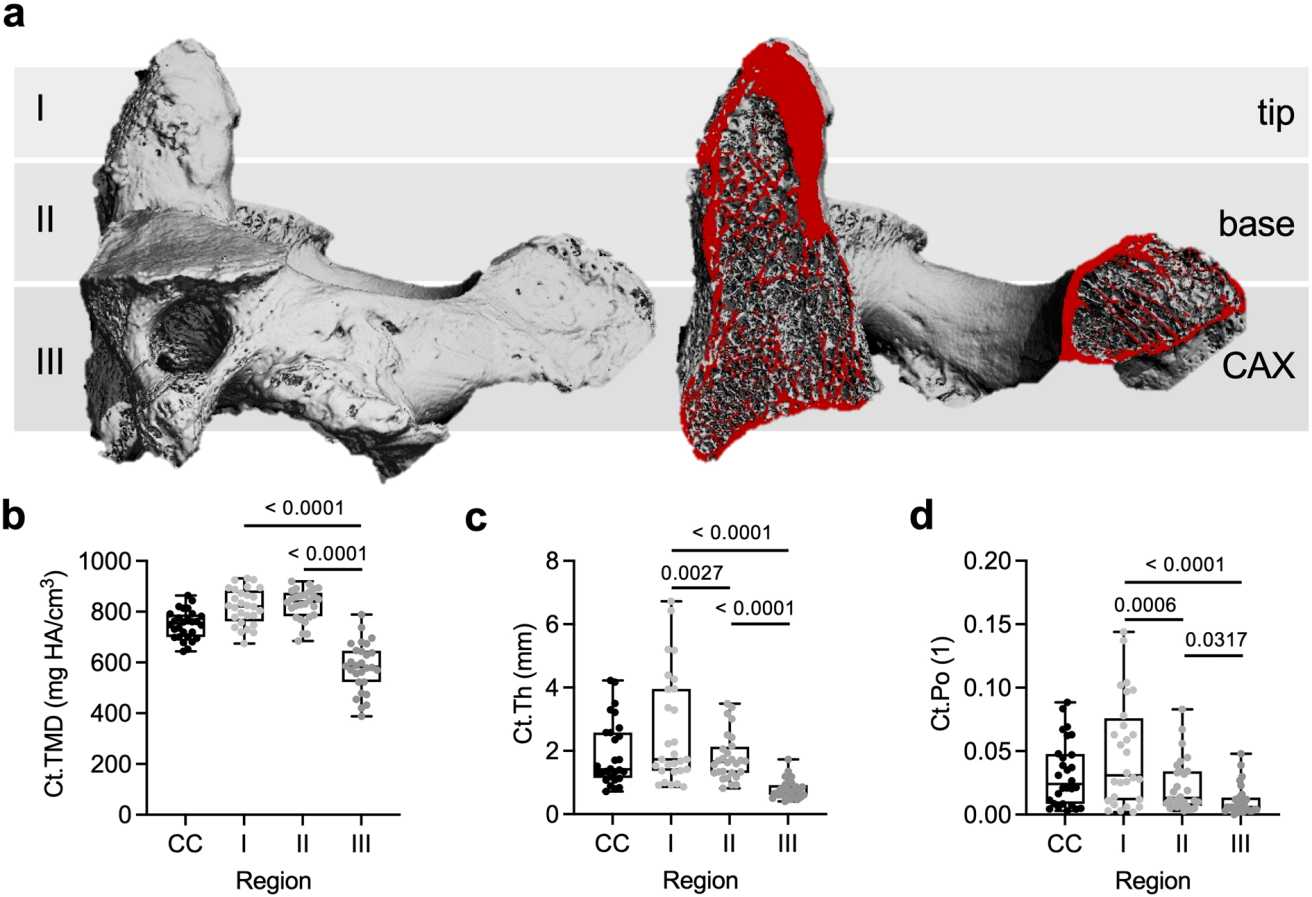
Region-specific cortical microarchitecture of the axis assessed by HR-pQCT. **a** Definition of the measurement regions in a 3D-segmented representative specimen (left) with red colored marking of a virtual cut section (right). Note the labeling of the measurement regions, *i.e.*, tip, base, CAX, for HR-pQCT analysis (Zone I-III). **b** Quantification of the cortical tissue mineral density in zones I-III as well as the complete dens. **c** Quantification of the cortical thickness. **d** Quantification of the cortical porosity in all regions. Significant differences are indicated by exact *p*-values

A total of *n* = 28 (14 male and 14 female) human axis specimens was analyzed. The mean age at death was 80.8 ± 13.9 years. Compared with clinical cases, no significant difference could be identified with respect to age (*p* = 0.738). Results of trabecular and cortical parameters obtained via HR-pQCT are presented in Table 2. Determinants of the cortical microarchitecture and mineralization are additionally presented in Fig. 3. Zone III presented with lower cortical TMD compared to zones I (*p* < 0.001) and II (*p* < 0.001) (Fig. 3b). Cortical thickness (Ct.Th) decreased from tip to corpus of the axis (zone I *vs*. II *p* = 0.03; zone I *vs.* III *p* < 0.001; zone II *vs*. III *p* < 0.001) (Fig. 3c). Similarly, Ct.Po decreased continuously from the tip to the corpus (zone I *vs*. II *p* < 0.001; zone I *vs.* III *p* < 0.001; zone II *vs*. III *p* = 0.032) (Fig. 3d).

**Table 2:** Microarchitecture and mineral density of trabecular and cortical bone in the axis obtained by high resolution peripheral quantitative computed tomography in autopsy specimens

Parameter	CC	Zone I (tip)	Zone II (base)	Zone III (CAX)
BV/TV (1)	0.26 ± 0.10	0.49 ± 0.20*^,§^	0.27 ± 0.10^$^	0.17 ± 0.08
Tb.N (1/mm)	1.03 ± 0.19	1.28 ± 0.26*^,§^	1.06 ± 0.18	1.11 ± 0.22
Tb.Th (mm)	0.36 ± 0.11	0.54 ± 0.30*^,§^	0.34 ± 0.08^$^	0.22 ± 0.03
Tb.Sp (mm)	0.98 ± 0.23	0.76 ± 0.26*^,§^	0.95 ± 0.22	0.92 ± 0.21
Conn.D (1/mm^3^)	1.83 ± 0.66	1.38 ± 1.00^§^	1.65 ± 0.57^$^	2.24 ± 1.17
SMI (1)	1.40 ± 0.77	− 1.63 ± 2.48*^,§^	1.01 ± 0.88^$^	1.98 ± 0.71
DA (1)	1.14 ± 0.06	1.26 ± 0.19	1.19 ± 0.09	1.20 ± 0.10
vBMD (mgHA/cm^3^)	205.7 ± 73.3	378.4 ± 169.1*^,§^	213.4 ± 73.4^$^	148.0 ± 56.0
TMD (mgHA/cm^3^)	748.6 ± 43.0	760.9 ± 72.9^§^	748.3 ± 37.5^$^	696.6 ± 38.2
Ct.Th (mm)	1.90 ± 1.03	2.61 ± 1.74*^,§^	1.79 ± 0.74^$^	0.78 ± 0.30
Ct.Po (1)	0.032 ± 0.026	0.048 ± 0.042*^,§^	0.023 ± 0.021^$^	0.011 ± 0.012
Ct.vBMD (mgHA/cm^3^)	724.4 ± 54.6	780.3 ± 75.8*^,§^	809.3 ± 62.8^$^	574.6 ± 90.8
Ct.TMD (mgHA/cm^3^)	748.7 ± 55.9	819.8 ± 70.5^§^	828.2 ± 61.1^$^	582.0 ± 97.0

*CC* complete cylinder, *BV* bone volume, *TV* tissue volume, *Tb.N* trabecular number, *Tb.Th* trabecular thickness, *Tb.Sp* trabecular separation, *Conn.D* connectivity density, *SMI* structure model index, *DA* degree of anisotropy, *vBMD* volumetric bone mineral density, *TMD* tissue mineral density, *Ct.Th* cortical thickness, *Ct.Po* cortical porosity, *mgHA* milligram hydroxyapatite

Data is shown in mean ± standard deviation

*Zone I vs. II = *p* < 0.05, ^§^zone I vs. III = *p* < 0.05, ^$^zone II vs. III = *p* < 0.05

**Fig. 4:**
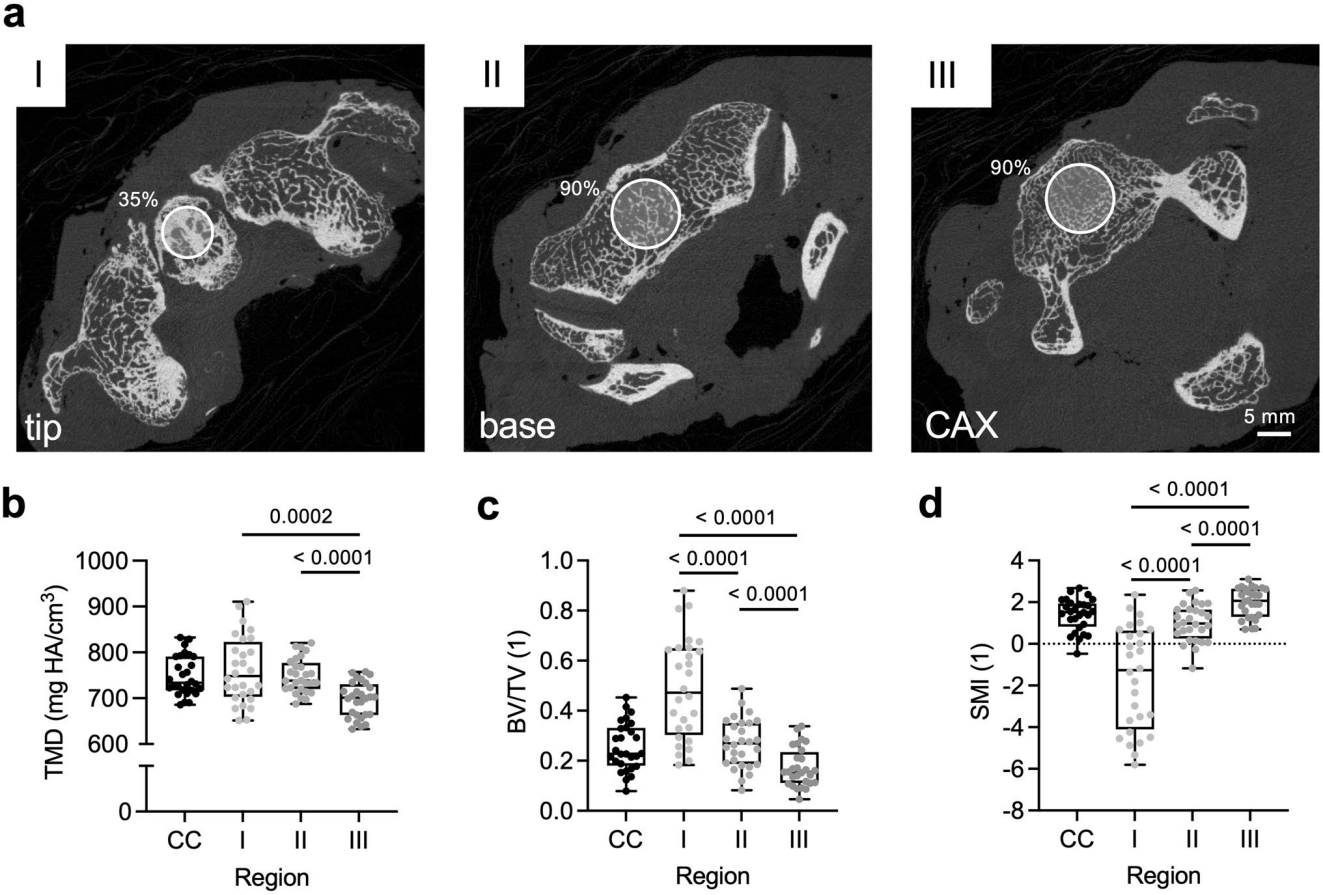
Region-specific trabecular microarchitecture of the axis assessed by HR-pQCT. **a** Axial slices in the three measurement regions (Zones I-III, voxel size of 61 µm each) with circles indicating the volume of interest. The diameter was determined as 35% and 90% of the maximum DAX diameter at the transition to the superior articular process. **b** Quantification of the trabecular tissue mineral density in zones I-III as well as the complete cylinder. **c** Quantification of the bone volume per tissue volume. **d** Quantification of the structure model index indicating plate- and rod-like trabecular structures. CC–complete cylinder. Significant differences are indicated by exact *p*-values

Analysis of the trabecular compartment (Fig. 4a) revealed lower TMD in zone III compared to the other two regions (zone I *vs*. II *p* = 0.410; zone I *vs*. III *p* < 0.001; zone II *vs*. III *p* < 0.001) (Fig. 4b). Moreover, a decrease in BV/TV was observed between zones I to III (zone I *vs*. II *p* < 0.001; zone I *vs*. III *p* < 0.001; zone II *vs*. III *p* < 0.001) (Fig. 4c). Analogously, Tb.Th decreased along zones I-III (zone I *vs*. II *p* < 0.001; zone I *vs*. III *p* < 0.001; zone II *vs*. III *p* < 0.001). While Tb.Sp was lower in zone I compared to both other zones (zone I *vs*. II *p* < 0.001; zone I *vs.* III *p* = 0.004; zone II *vs*. III *p* = 0.695), zone I showed the highest Tb.N compared to zone II and III (zone I *vs.* II *p* < 0.001; zone I *vs*. III *p* = 0.006; zone II *vs*. III *p* = 0.243). Conn.D in zone III was higher than in zone I and II (zone I *vs*. II *p* = 0.354; zone I *vs*. III *p* = 0.020; zone II *vs*. III *p* = 0.011). The mean SMI in zone I was negative and showed lower values than zone II and III (zone I *vs.* II *p* < 0.001; zone I *vs*. III *p* < 0.001; zone II *vs*. III *p* < 0.001), showing a plate-like, sclerotic configuration of trabeculae in the apex of the DAX, which changes to rod-like trabeculae caudally (Fig. 4d). DA did not vary between the three analyzed regions (zone I *vs*. II *p* = 0.169; zone I *vs*. III *p* = 0.334; zone II *vs.* III *p* = 0.734).

### Sex Differences

Although sex differences in trabecular and cortical parameters were observed (Supplementary Fig. 1, 2), the effect of age and sex on region-specific trends was largely negligible (Supplementary Table 1, 2). More specifically, differences between zones remained unaffected; however, when females were evaluated separately, similar Conn.D in all zones was observed, unlike in males and the overall population. Similarly, Ct.Po only differed between zones in male specimens.

## Discussion

In the present study, the bone microarchitecture of the axis was analyzed using ex vivo HR-pQCT. The results were correlated with CT data from patients to derive implications for the occurrence and care of axis fractures. The major findings were: (1) CT-based apparent densities in zone I were higher than in zone II and III, mainly independent of fracture occurrence. (2) Cortical and trabecular microarchitecture parameters decreased from zone I in the tip of the DAX to zone III in the CAX, while trabecular separation was lowest and trabecular number highest at the apex accordingly. (3) The trabecular and cortical tissue mineral density was similar at the base and tip, while lowest at the CAX. (4) The SMI indicated a plate-like and dense, sclerotic trabecular microarchitecture in the tip of the DAX transforming into a highly cross-linked rod-like trabecular microarchitecture in the CAX.

In clinical cases, we demonstrated lower apparent density in zone II and III than in zone I, irrespective of fracture status. In addition, the sagittal plane of zone II in the fracture group, but not the non-fracture group, showed a higher apparent density than the sagittal plane in zone III. This difference could be explained by the higher apparent density in the fracture area due to the occurrence of hematoma and the impaction of trabeculae. Although no differences in individual zones were detected between fractured and non-fractured patients, there was a pronounced regional heterogeneity in both the non-fracture and the fracture group with lower apparent density in zones II and III compared to zone I. This could be a correlate of reduced bone quality at the base of the DAX and in the CAX.

As it is known that clinical CT scans provide only a rough estimate of BMD, since the apparent density in HU cannot be directly translated into BMD, the bone quality and microarchitecture of the axis was further analyzed experimentally using HR-pQCT on full bone specimens. To our best knowledge, this is the very first study to analyze the bone microarchitecture of the axis via HR-pQCT in a comparable sample size of *n* = 28, following a clinically relevant fracture classification and correlating the findings to clinical groups of patients with and without fractures of the axis. We suggest that the decreasing cortical and trabecular microarchitecture from the tip of the DAX to the CAX is a major factor influencing fracture susceptibility. Notably, similar analyses have been previously performed by our group in other skeletal regions such as the distal fibula, inferring fracture mechanisms based on regional heterogeneity in local bone microarchitecture [24].

In 1994, Amling et al*.* analyzed the microarchitecture of the axis histologically in *n* = 22 autopsy specimens with a mean age of 50 years [15]. For analysis, they also chose a division of the axis into three zones following the classification of Anderson and D'Alonzo. They found a BV/TV of 20% in the CAX, 10% in the base of the DAX, and 26% in the odontoid process. The BV/TV was significantly lower in the base of DAX compared to the other zones. Moreover, the trabecular pattern factor, a parameter that indirectly accounts for inter-trabecular connections by determining the relation of convex and concave surface patterns [25], was determined in these regions, with the worst trabecular connection detected at the base of the DAX. The group concluded the presence of a region of least resistance in the base of the DAX due to lower bone mass and weaker bone microarchitecture [15]. In a subsequent study, Amling et al. provided additional data from *n* = 11 autopsy specimens with known osteoporosis and deduced an increased risk for fractures and subsequent non-unions of the dens in the osteoporotic bone due to impaired bone quality in the base of the DAX. Although providing important insights, these previous analyses were performed only two-dimensionally in the sagittal plane [15, 16]. Our data offer insights into the bone quality and microarchitecture in the corresponding regions of the axis three-dimensionally. From the current data, it can be concluded that in zone II, which corresponds to the base of the DAX, and in zone III in the CAX, which may include a residual subdental synchondrosis, the trabecular bone is weaker than in zone I in the apex of the DAX due to a lower bone mass with less and thinner trabeculae. Further, the differences in BMD indicated higher mineralization within the two cranial zones supporting this assumption. Regarding the microarchitecture of the trabecular bone, the SMI and the Conn.D indicated a dense, sclerotic, and plate-like structure at the tip of the dens, while the CAX presented with high interconnectivity.

Previous studies have shown that local bone mass and microarchitecture have a major influence on the occurrence of fractures [26–31]. By analyzing bone specimens from lumbar vertebrae via image-guided failure assessment under microtomographic imaging, it was shown that a decrease in BV/TV is accompanied by an increased fracture probability and that especially the combination of low BV/TV and Conn.D indicates the weakest bone region. The authors concluded that the weakest region within a bone structure may be crucial for fracturing the whole complex and that the above mentioned structural parameters are crucial for identifying these weak regions [26]. Furthermore, a positive correlation of a low Tb.N and a high Tb.Sp with the occurrence of vertebral fractures has been shown in the past [30]. Although no biomechanical studies with combined consideration of high-resolution microarchitecture of the axis have been performed to date, previous observations suggest that microarchitecture plays a major role in influencing the occurrence of fractures. However, the occurrence of fractures of the axis must be considered in a more differentiated manner and cannot be attributed to a single aspect such as bone quality. Both anatomical variations, *i.e.*, bone structure, bone composition, joints, and ligaments, and trauma mechanism are decisive. A recent biomechanical study investigated the influence of bone density on the occurrence of axis fractures indicating an increased fracture risk with low BMD while the direction of the applied loading showed little influence [31]. Nevertheless, especially the articular connection between the anterior arch of the atlas and the DAX seems likely to be relevant for the occurrence of the DFTII. While fractures of the axis in young patients often arise in the context of high-energy traumas, a typical trauma mechanism in geriatric patients is a frontal head impact causing a reclination in the upper cervical spine. It is likely that this anatomical feature and its resulting high mechanical moments accompanied by a low BMD in the base of the DAX determines the high fracture susceptibility for DFTII.

Our data show that in the extension of the DAX caudally into the CAX, Ct.Th, BV/TV, and trabecular as well as cortical TMD continued to decrease, whereas Amling et al. postulated an increase in BV/TV [15]. These differences might be due to methodological discrepancies (*i.e.*, 2D *vs.* 3D analysis) and age differences of the groups investigated. Due to the obvious difficulty in obtaining samples, there are no studies that have investigated the trabecular bone of the axis using HR-pQCT, in a similarly large collective to date. Recently, Wang et al. analyzed *n* = 5 dry bone samples with a mean age of 52 years [23]. Four volumes of interest (VOI) were analyzed within the trabecular bone of the DAX. VOI I was defined in the tip of the DAX, VOI II in the neck of the DAX, VOI III in the body of the DAX, and VOI IV in the base of the DAX. In this previous study, a higher bone mass, and thicker and more numerous trabeculae were determined in VOI I compared to VOI IV, whereas trabecular separation was lower in VOI I than in VOI IV [23]. While these findings obtained in a small collective of *n* = 5 specimens are generally consistent with our data, an analysis of CAX was not performed in the study by Wang et al. [23]. In our view, an additional analysis of CAX seems essential. The classification most commonly used in clinical practice to classify fractures of the axis, regardless of the gaps that certainly exist, is the classification according to Anderson and D'Alonzo [1]. DFTII followed by DFTIII occur most frequently, whereas DFTI are very rare [2]. While DFTII occur at the base of the DAX, DFTIII occur within the cylinder that results from an extension of the DAX caudally into the CAX. Furthermore, in this cylinder lies the screw trajectory for anterior screw fixation, frequently performed for the surgical treatment of a DFTII, according to Böhler et al.[11]. For these reasons, we considered an analysis of the bone microarchitecture in zones I–III based on the classification of Anderson and D'Alonzo to be suitable, to derive clinical implications.

The following clinical implications emerge from our and previous results: The base of the DAX is prone to fracture based on the combination of low bone mass, bone mineralization, and trabecular microarchitecture [15–17, 20, 23]. In addition, the subdental trabecular bone represents a biomechanical weak point within the axis. On the one hand, this may account for the frequent occurrence of DFTIII in addition to DFTII. On the other hand, our data provide a possible explanation as to why the typical cut-out of screws after anterior screw fixation often occurs in zones II and III, which was also supported by the data described by the CT-based apparent densities in the clinical part of the study. Therefore, we consider anterior screw fixation to bear a high risk for failure, especially in geriatric patients [9, 12, 13]. However, due to the important advantages of the technique compared to alternative or non-surgical procedures, such as the low invasiveness and the preserved rotational ability between atlas and axis, we consider the establishment of alternative osteosynthesis procedures for the placement of lag screws in the DAX with a separate anchorage in the CAX, such as osteosynthesis plates, essential to increase the quality of care and safety of elderly patients with fractures of the axis. These aspects should be further investigated in additional studies.

Limitations of our study include that only non-fractured axis specimens could be analyzed by HR-pQCT. Thus, no direct comparison of microarchitecture between non-fracture and fracture groups could be made, which should be performed in the future. Although full autopsy allowed us to exclude conditions that locally affect skeletal microarchitecture (e.g., tumors), the presence of osteoporosis could not be determined by established methods. Nevertheless, the autopsy specimens offered us a unique opportunity to perform the high-resolution HR-pQCT examination, which is usually limited to distal bones (radius, tibia) [32] and cannot be performed on the cervical spine in the clinical setting. Another limitation is that HR-pQCT measurements of the specimens were performed with a resolution of nearly 15–30% (depending on the region) of the trabecular thickness. Hence, the partial volume effect might have an influence on the mineralization results of this study. However, additional calculation showed similar correlation coefficients between TMD and thickness in the thinnest and thickest structures, indicating a negligible influence (*p* = 0.76). Another limitation of our study is that the analysis was limited to a geriatric patient population. The resulting narrow age range prevented a meaningful analysis of age-related bone loss, and thus the presumed influence of age on fracture risk or screw loosening.

In conclusion, the axis is characterized by a decreasing cortical and trabecular microarchitecture from the tip of the DAX to the CAX, indicating inferior bone quality in this region. These findings may partly explain the clinical observation that zones II and III, the base of the DAX and in the CAX, depicture sites with a higher fracture susceptibility than zone I, the tip of the DAX. While non-bony anatomical structures and trauma mechanism certainly also play a critical role regarding fracture occurrence, the reduced bone quality in zones II and III could be a risk factor for implant loosening after anterior screw fixation. In order to ensure safe osteosynthesis of axis fractures, improved, additional anchorage of the implants in zones II or III, e.g., by osteosynthesis plates, might be indicated, especially in aged patients with limited bone status.

## Supplementary Information

Below is the link to the electronic supplementary material.

Supplementary file1 (DOCX 1770 kb)
