# Supplementary material for: Analysis of Three-Dimensional Bone Microarchitecture of the Axis Exposes Pronounced Regional Heterogeneity Associated with Clinical Fracture Patterns
Source: Calcif Tissue Int. 2023 Feb 24;112(5):563–72. doi: 10.1007/s00223-023-01070-7 (PMC10106346; doi:10.1007/s00223-023-01070-7)
Supplement: Supplementary file 1 — Supplementary file1 (DOCX 1770 kb) [file 223_2023_1070_MOESM1_ESM.docx]

**Supplementary Material**

**Analysis of three-dimensional bone microarchitecture of the axis exposes pronounced regional heterogeneity associated with clinical fracture patterns**

**Supplementary Figure 1:** Sex-specific microstructural analysis of the cortical compartment in the DAX. (a-d) Quantification of Ct.Th. Ct.Po, Ct.vBMD, and Ct.TMD in both female (red) and male (blue) specimens. RM ANOVA and Tukey post-hoc testing was used in all panels. a – significant difference to the same region in female specimens.

**Supplementary Figure 2:** Sex-specific microstructural analysis of the trabecular compartment in the DAX. (a-i) Quantification of BV/TV, Tb.N, Tb.Th, Tb.Sp, Conn.D, SMI, DA, vBMD, and TMD in both female (red) and male (blue) specimens. RM ANOVA and Tukey post-hoc testing was used in all panels. a – significant difference to the same region in female specimens. CC – complete cylinder.

**Supplementary Table 1:** Dependency of sex and age on region-specific differences in cortical bone of the dens axis.

|  |  | P_region_ (I-II) | p_region_ (I-III) | p_region_ (II-III) | Partial Eta Squared |
| --- | --- | --- | --- | --- | --- |
| Ct.Th | ANOVA | 0.003 | < 0.001 | < 0.001 | 0.520 |
|  | ANCOVA (sex) | 0.002 | < 0.001 | < 0.001 | 0.558 |
|  | ANCOVA (age) | < 0.001 | < 0.001 | < 0.001 | 0.902 |
| Ct.Po | ANOVA | < 0.001 | < 0.001 | **0.032** | 0.424 |
|  | ANCOVA (sex) | < 0.001 | < 0.001 | 0.034 | 0.490 |
|  | ANCOVA (age) | 0.001 | 0.001 | **0.117** | 0.809 |
| Ct.vBMD | ANOVA | **0.014** | < 0.001 | < 0.001 | 0.782 |
|  | ANCOVA (sex) | 0.007 | < 0.001 | < 0.001 | 0.815 |
|  | ANCOVA (age) | **0.061** | < 0.001 | < 0.001 | 0.952 |
| Ct.TMD | ANOVA | 0.609 | < 0.001 | < 0.001 | 0.819 |
|  | ANCOVA (sex) | 0.609 | < 0.001 | < 0.001 | 0.834 |
|  | ANCOVA (age) | 0.891 | < 0.001 | < 0.001 | 0.959 |

P-values of RM ANOVA and RM ANCOVA (covariates “sex” and “age”) testing including effect size (Partial Eta Squared) with subsequent Tukey post-hoc tests. Differences of RM ANCOVA and RM ANOVA testing are highlighted in bold.

**Supplementary Table 2:** Dependency of sex and age on region-specific differences in trabecular bone of the dens axis.

|  |  | p_region_ (I-II) | p_region_ (I-III) | p_region_ (II-III) | Partial Eta Squared |
| --- | --- | --- | --- | --- | --- |
| BV/TV | ANOVA | < 0.001 | < 0.001 | < 0.001 | 0.766 |
|  | ANCOVA (sex) | < 0.001 | < 0.001 | < 0.001 | 0.786 |
|  | ANCOVA (age) | < 0.001 | < 0.001 | < 0.001 | 0.942 |
| Tb.N | ANOVA | < 0.001 | 0.006 | **0.243** | 0.366 |
|  | ANCOVA (sex) | < 0.001 | 0.002 | 0.208 | 0.419 |
|  | ANCOVA (age) | < 0.001 | 0.018 | **0.047** | 0.784 |
| Tb.Th | ANOVA | < 0.001 | < 0.001 | < 0.001 | 0.543 |
|  | ANCOVA (sex) | < 0.001 | < 0.001 | < 0.001 | 0.579 |
|  | ANCOVA (age) | 0.003 | < 0.001 | < 0.001 | 0.845 |
| Tb.Sp | ANOVA | < 0.001 | 0.004 | 0.695 | 0.299 |
|  | ANCOVA (sex) | < 0.001 | 0.002 | 0.694 | 0.318 |
|  | ANCOVA (age) | < 0.001 | 0.004 | 0.217 | 0.826 |
| Conn.D | ANOVA | 0.354 | 0.020 | 0.011 | 0.209 |
|  | ANCOVA (sex) | 0.199 | < 0.001 | 0.003 | 0.333 |
|  | ANCOVA (age) | 0.209 | 0.005 | 0.011 | 0.692 |
| SMI | ANOVA | < 0.001 | < 0.001 | < 0.001 | 0.711 |
|  | ANCOVA (sex) | < 0.001 | < 0.001 | < 0.001 | 0.771 |
|  | ANCOVA (age) | < 0.001 | < 0.001 | < 0.001 | 0.924 |
| DA | ANOVA | 0.169 | 0.334 | 0.734 | 0.085 |
|  | ANCOVA (sex) | 0.177 | 0.344 | 0.742 | 0.086 |
|  | ANCOVA (age) | 0.109 | 0.352 | 0.688 | 0.315 |
| vBMD | ANOVA | < 0.001 | < 0.001 | < 0.001 | 0.716 |
|  | ANCOVA (sex) | < 0.001 | < 0.001 | < 0.001 | 0.741 |
|  | ANCOVA (age) | < 0.001 | < 0.001 | < 0.001 | 0.923 |
| TMD | ANOVA | 0.410 | < 0.001 | < 0.001 | 0.427 |
|  | ANCOVA (sex) | 0.325 | < 0.001 | < 0.001 | 0.479 |
|  | ANCOVA (age) | 0.142 | < 0.001 | 0.001 | 0.849 |

P-values of RM ANOVA and RM ANCOVA (covariates “sex” and “age”) testing including effect size (Partial Eta Squared) with subsequent Tukey post-hoc tests. Differences of RM ANCOVA and RM ANOVA testing are highlighted in bold.
